# Treerunners, cryptic lizards of the *Plica plica* group (Squamata, Sauria, Tropiduridae) of northern South America

**DOI:** 10.3897/zookeys.355.5868

**Published:** 2013-11-25

**Authors:** John C. Murphy, Michael J. Jowers

**Affiliations:** 1 Science and Education, Field Museum of Natural History, 1400 S. Lake Shore Drive, Chicago, IL 60605 USA; 2 CSIC (Consejo Superior de Investigaciones Científicas), Departamento de Etología y Conservación de la Biodiversidad, Estación Biológica de Doñana, C/ Américo Vespucio, s/n (Isla de la Cartuja), 41092 Sevilla, España

**Keywords:** Arboreal lizards, Iguania, Neotropics, new species, systematics

## Abstract

The arboreal, Neotropical lizard *Plica plica* (Linnaeus, 1758) has been long considered a widespread species with a distribution east of the Andes. A preliminary examination of 101 specimens from about 28 locations mostly north of the Amazon suggests that *Plica plica* is a cryptic species complex with taxa that can be distinguished on the basis of the number of scale rows at mid-body; the arrangement, shape and ornamentation of scales on the snout; the number of lamellae on the fourth toe; the number of subocular plates; as well as other commonly used external morphological traits. The allopatric species discussed here are concordant with northern South American geography. *Plica plica* (Linnaeus, 1758) is associated with the Guiana Shield (Suriname, Guyana and Venezuela). A second species, *P. caribeana* **sp. n**. is associated with the Caribbean Coastal Range of Venezuela including Trinidad and Tobago. A third, distinctive species, *P. rayi* **sp. n**. is associated with the middle Orinoco at the eastern edge of the Guiana Shield. Two other species, *P. kathleenae* **sp. n.** and *P. medemi* **sp. n.**, each based upon a single specimen, one from the Sierra Acarai Mountains of Guyana, and the other from southern Meta, Colombia are described. In addition to morphological analyses, we sequenced 12S and 16S rDNA gene fragments from one *Plica plica* from Trinidad to assess its relationship and taxonomy to other mainland *Plica* cf. *plica*. The results suggest *Plica caribeana* **sp. n.** likely diverged prior to the separation of Trinidad from northern Venezuela. Isolation in the Caribbean Coastal Range during its rapid uplift in the late Miocene, combined with a marine incursion into northern Venezuela may have contributed to their genetic divergence from other populations.

## Abstract (es)

La especie arbórea de lacértido Neotropical *Plica plica* ha sido considerada por mucho tiempo una especie ampliamente distribuida al este de Los Andes. Un examen preliminar de 101 especímenes de más de 28 localidades, mayoritariamante del norte del Amazonas, sugiere que *Plica plica* es un complejo de especies crípticas con taxa que pueden ser distinguidos principalmente por el número de filas de escamas en el centro del cuerpo; la disposición, la forma y ornamentación de las escamas en el hocico; el número de lamellas en el cuarto dígito del pie; el número de placas suboculares; así como por otras características morfológicas comúnmente empleadas. Las especies alopátricas aquí discutidas concuerdan con la geografía del norte de Sur América. *Plica plica* (Linnaeus, 1758) está asociada al escudo Guyanés (Surinam, Guyana y Venezuela). Una segunda especie, *P. caribeana* **sp. n.** está asociada a la zona costera montañosa de Venezuela, incluyendo Trinidad y Tobago. Una tercera distintiva especie, *P. rayi* **sp. n.** está asociada al centro del Orinoco. Dos otras especies, *P. kathleenae* **sp. n.** y *P. medemi* **sp. n.** están descritas en base a un solo ejemplar, una para las montañas de la Sierra Acarai de Guyana, y la otra para el sur de Meta (Colombia) respectivamente. Algunas especies amazónicas pueden ser simpátricas basándose en las localidades de los especímenes de museos. Además de análisis morfológicos, secuenciamos una fracción del gen 12S y 16S rDNA de un especimen de *Plica plica* de Trinidad para investigar su relación y taxonomía con *Plica* cf. *plica* del continente. Los resultados sugieren que *Plica plica* de Trinidad y de la cordillera costera de Venezuela probablemente divergieron antes de la separación de Trinidad del norte de Venezuela. El aislamiento en la región de la cordillera costera de Venezuela durante el rápido levantamiento en el Mioceno tardío, combinado con una posible inclusión marina en el norte de Venezuela, probablemente contribuyó aún más a la divergencia genética de otras poblaciones.

## Introduction

Tropidurid lizards are usually scansorial, dwelling on vertical rocks and tree trunks with morphology that includes strongly keeled scales that contribute to their cryptic appearance. The family currently holds eight genera and about 117 species. Townsend et al. (2011) suggest that family last shared and ancestor with the other clades of Neotropical iguanians about 65 million years ago (MYA).

The tropidurid genus *Plica* Gray, 1831 (treerunners) currently contains four species restricted to South America east of the Andes. Two of these are relatively widespread (*Plica plica* and *Plica umbra*). The other two species are associated with Pantepuis. *Plica lumaria* is known only from southern Venezuela’s Guaiquinima Tepui (Donnelly and Myers 1991), and *Plica pansticta* (Myers and Donnelly 2001) from the Yutajé–Corocoro massif of Amazonas, Venezuela. Treerunnersare diurnal, medium sized, sit in the open on vertical surfaces, and are often in small colonies that include adults of both sexes and juveniles. The sounds they make scurrying on the bark of trees or rock outcrops draws attention to their presence and thus, they are common in museum collections.

In a review of *Plica*, Etheridge (1970) restricted the type locality for *Lacerta plica* Linnaeus to the vicinity of Paramaribo, Suriname, designating NRM.112 as the lectotype. Hoogmoed (1973) further restricted the locality to the confluence of the Cottica River and Perica Creek, Suriname. *Plica plica* is known from the countries of Bolivia, Brazil, Colombia, Ecuador, Guyana, Peru, Suriname, and Venezuela, as well as the islands of Trinidad and Tobago, and including the Bocas Island group (Boos 1984; Avila-Pires 1995; Murphy and Downie 2012). Additionally, two specimens collected in the 19^th^ century in the British Museum have the locality data “Grenada” (Henderson and Murphy 2012).

**Figure 1.**
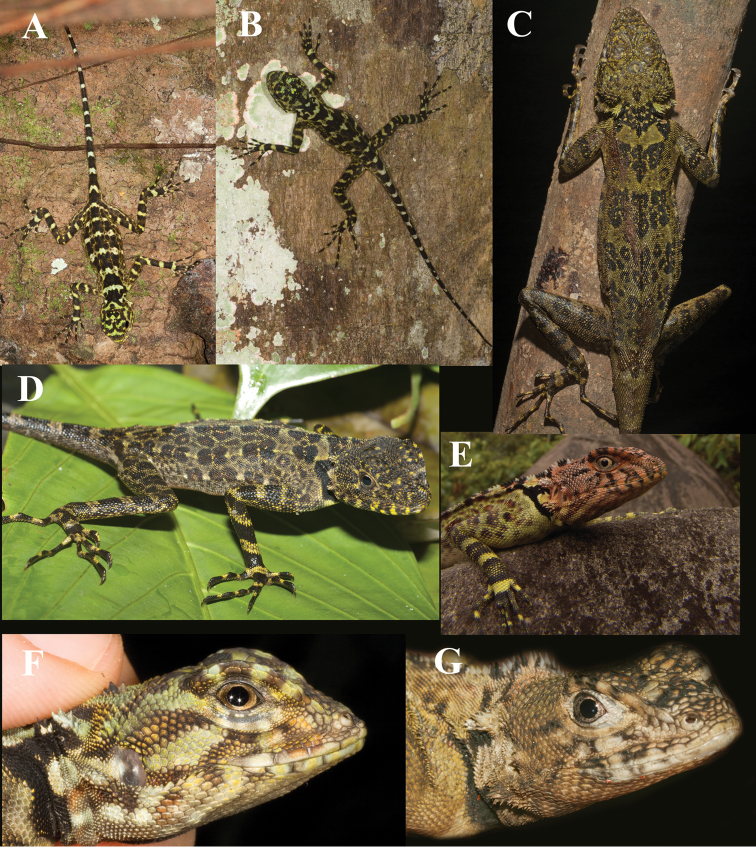
A composite of photographs of lizards in the *Plica plica* Group from various locations: **A** and **F** Tiputini Biodiversity Station, Napo, Ecuador, Ryan Sawby **B** Santa Cruz, Río Mazán near Iquitos (Iquitos, Loreto, Peru), Tobias Eisenberg **C** and **D** Amaila Falls, Guyana, Pedro Bernardo **E** La Laja, Sierra de Lema, 490 m., Estado Bolivar, Venezuela, César Luis Barrio Amorós **G** Cuesa River, Trinidad, JCM.

Of the four species of *Plica* currently recognized only *Plica umbra* lacks tufts of spines on the neck; and it has 43–69 scales around mid-body (Avila-Pires 1995). *Plica lumaria* is black, the superciliaries are directed laterally, it lacks clusters of spines on the fold below the auditory meatus, and it has 141–156 rows of scales around mid-body and 27–33 lamellae under the fourth toe (Donnelly and Myers 1991). *Plica pansticta* has 143–164 scales around mid-body and 31–39 lamellae under the fourth toe (Myers and Donnelley 2001). However, *Plica plica* (sensu Etheridge 1970) has 92–202 scales around mid-body; 21–35 lamellae on the fourth finger and 28–45 lamellae on the fourth toe. The polytypic *Plica plica* has been the subject of ecological (Vitt 1991; Brandt and Navas 2011), morphological (Kohlsdorf et al. 2007; Kohlsdorf et al. 2008), and phylogenetic studies (Frost 1992; Harvey and Gutberlet 2000; Frost et al. 2001). Figure 1 shows *Plica plica* from various localities and variation in their overall appearance. Etheridge (1970) seemed to be aware of at least some of the variation. He wrote:

The most deviant sample I have seen is from Puerto Ayachuco (sic) on the Orinoco River, between Venezuela and Colombia. Specimens from this locality differ from others in having a more acuminate snout, larger dorsal snout scales, slightly smaller dorsal body scales, less well developed lateral neck spines, and a less densely spotted throat in males. Specimens from the vicinity of Esmeralda, Venezuela also have large dorsal snout scales but are otherwise typical. Specimens from northern Venezuela, Trinidad, and the Guianas appear to reach the greatest maximum size, and because of greater development of scale mucrons have overall a more spiny appearance.

The intent of this work is not to revise the entire species group but rather to present morphological and molecular evidence that *Plica plica* is a complex of cryptic species, examine the availability of previously proposed names, resolve some of the species that occur in northern South America and suggest avenues for future investigations.

## Methods and materials

### Molecular methods

Individual DNA was extracted using the Chelex extraction process (Bio-Rad®, Hercules, 12 CA, U.S.A; Walsh et al. 1991) following slight protocol modifications. Muscle tissue (2 mm^3^) was cut thoroughly and incubated for 2h at 57°C in 160 μl of 5% Chelex with 40 μl Proteinase K to increase tissue digestion. Following the incubation period it was heated at 100°C for 15 minutes. After a 4 min centrifugation at 12500 rpm, all supernatant was transferred into a 1.5 ml tube. We aimed to amplify a fraction of the mitochondrial 12S and16S rDNA genes with mitochondrial vertebrate universal primers to compare with other *Plica plica* sequences available in Genbank. For each PCR, the 20 µl PCR volume contained approximately 50 ng DNA, 200 µM of each dNTP, 0.15 µM of each primer, 2 µl 10X Buffer, 0.8 µl Mgcl_2_ and 0.1 unit of taq polymerase (QIAGEN). The thermal cycle profile was as follows: an initial denaturation step of 2min at 94°C; 35 cycles of denaturation at 30s at 94°C, annealing for 30s at 52°C and extension for 45s at 72°C; and a final extension for 5min at 72°C. Following the PCR, excess primers and dNTPs were removed using enzymatic reaction of *Escherichia coli* Exonuclease I, Antartic phosphatase and Antartic phosphatase buffer (all New England Biolabs). Sequencing was carried out in both directions using the BigDye^®^ Terminator v1.1 cycle sequencing kit (Applied Biosystems) according to the manufacturer’s instructions. Labelled fragments were resolved on an automated A3130*xl* genetic analyzer (Applied Biosystems). The primers for the12S rDNA: 12SA 5´- AAACTGGGATTAGATACCCCACTAT- 3´, 12SB 5´- GAGGGTGACGGGCGGTGTGT-3´ Kocher et al. (1989) and 16S rDNA: 5´- GCCTGTTTATCAAAAACAT-3´, 16SH 5´- CCGGTCTGAACTCAGATCACGT- 3´ Palumbi et al. (1991) amplified approximately a 400 and 500 respectively base pair fraction (Genbank accessions: 12S rDNA; KU 895880, 16S rDNA; KU 895881). Templates were sequenced on both strands, and the complementary reads were used to resolve rare, ambiguous base-calls in Sequencher v.4.9. After removing PCR primers and incomplete terminal sequences, 337 and 378 base pairs of the 12S and 16S rDNA gene fraction, respectively (715 bp for both concatenated gene fractions) were available for analyses. Sequences were aligned in Seaview v.4.2.11 (Gouy et al. 2010) under ClustalW2 (Larkin et al. 2007) default settings. Nucleotide differences and *p*-uncorrected distances (%) analyses were calculated using MEGA v5 (Tamura et al. 2011).

In order to assess the phylogenetic relationships of *Plica plica* from Trinidad to the mainland, all *Plica plica* partial 12S and 16S rDNA sequences available from Genbank that could be aligned with ours were included in the alignment (Table 1). In addition, to root the phylogenetic tree, we employed as outgroup *Tropidurus oreadicus* and *Tropidurus insulanus*, members of a clade that is the sister to the clade containing *Plica* (Pyron et al. 2013).

**Table 1.** Combined matrices for the 12S rDNA and 16S rDNA gene fraction of *p*-uncorrected distances (below) and nucleotide substitutions (above). Specimen Genbank accessions: *Plica plica* (unknown); AB218961, *Plica plica* (Venezuela); 12S rDNA (L41431) 16S rDNA (L41482), *Plica plica* (Guyana?); 12S rDNA (AF362520), *Plica plica* (Brazil); 12S rDNA (EF615595) 16S rDNA (EF615664), *Tropidurus insulanus*; 12S rDNA (EF615596) 16S rDNA (EF615665), *Tropidurus oreadicus*; 12S rDNA (EF615597) 16S rDNA (EF615666).

	*Plica* (Tri)	*Plica* (unk)	*Plica* (Ven)	*Plica* (Guy)	*Plica* (Bra)	*T. insu*	*T. orea*
(1) *Plica plica* (Trinidad)	-	3	25	15	36	70	72
(2) *Plica plica* (unknown)	0.0043	-	22	13	33	71	73
(3) *Plica plica* (Venezuela)	0.0455	0.0400	-	6	15	49	51
(4) *Plica plica* (Peru)	0.0445	0.0386	0.0282	-	0	43	44
(5) *Plica plica* (Brazil)	0.0518	0.0475	0.0274	0.0000	-	71	74
(6) *Tropidurus insulanus*	0.1013	0.1027	0.0901	0.1284	0.1033	-	29
(7) *Tropidurus oreadicus*	0.1039	0.1053	0.0934	0.1313	0.1074	0.0417	-

The most appropriate substitution model for the Bayesian Inference (BI) analysis was determined by the Bayesian Information Criterion (BIC) in jModeltest v.0.1.1 (Posada 2008). The tree was constructed using the Bayesian Inference (BI) optimality criteria under the best fitting model (TIM2+I). MrBayes v.3.1.2 (Huelsenbeck and Ronquist 2001) was used with default priors and Markov chain settings, and with random starting trees. Each run consisted of four chains of 5,000,000 generations, sampled each 10,000 generations. A plateau was reached after few generations with 25% of the trees resulting from the analyses discarded as “burn in”.

### Morphological methods

**Figure 2.**
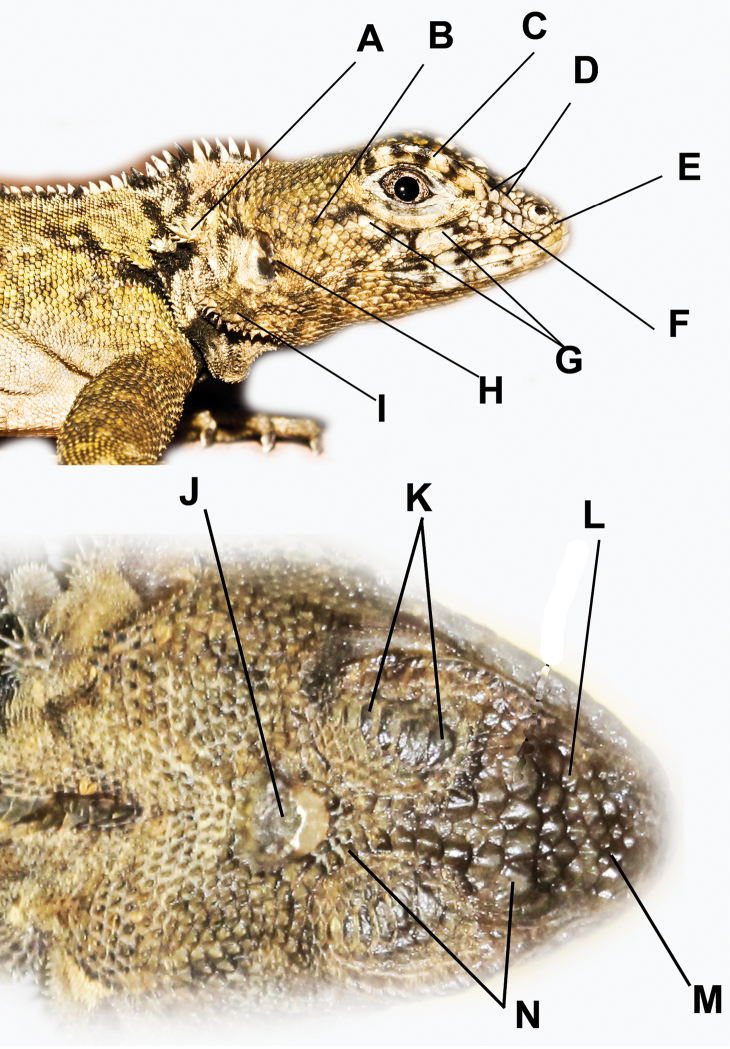
Head scale nomenclature used here. **A** Nape clusters or tufts of mucronate scales **B** Temporal scales **C** Superciliaries **D** Canthal(s) **E** Scale(s) between nasal and rostral **F** Loreals **G** Keeled suboculars **H** Cluster of spines on the anterior edge of the auditory meatus **I** Clusters (or tufts) of spines on the ventral flap of the auditory meatus **J** Occipitoparietal scale **K** Supraocular plates **L** Intercanthal scales **M** Internasal scales **N** Circumorbital scales.

We examined 101 museum specimens labeled *Plica plica* from: Brazil (n=8), Colombia (n=7), Ecuador (n=9), Guyana (n=2), Peru (n=25), Suriname (n=6), Trinidad (n=17), Venezuela (n=25), and Grenada (n=2). This was done to obtain a broad view of the variation within the species group, while focusing this paper on the Guiana Shield, the Coastal Ranges of northern Venezuela and Trinidad. Specimens examined from the area of study are listed in each species account; specimens examined but not assigned to a name are listed in Appendix 1. Scale counts and nomenclature follow that used by previous workers (Etheridge 1970; Hoogmoed 1973; Frost 1992; Avila-Pires 1995). Measurements of body and tail lengths were taken to the nearest 1 mm; scale measurements were taken to the nearest 0.1 mm and made with dial calipers. Values for paired head scales are given in left/right order. Note the terms spiny and mucronate are used interchangeably in reference to the well-developed keels on scales. Scale counts taken around mid-body were counted from one side of the dorsal crest to the other; ventral counts are scales between the ventrolateral folds. Photographs were taken with a Spot Digital Xplorer^TM^ camera fixed to a Leica compound microscope, and with a Cannon EOS Rebel^TM^ with a 100 mm macro lens. Statistical analysis was done with EXCEL-QI MACROS and DATA LAB 2.6. The PCA used 31 specimens with complete data, damaged specimens, or specimens with incomplete data were not included. Appendix 2 gives factor loadings for 25 meristic and morphometric traits. Institutional abbreviations used are given in the Acknowledgements. Abbreviations used: OUT – operational taxonomic units, PCA – principal component analysis, SAAM – position of spines on anterior margin of auditory meatus, SAB – scales around mid- body, SD – standard deviation, SRN – scales between nasal and rostral, STN – spiny tufts on nape, SVL – snout- vent length. Clarification of the head scale nomenclature used here is illustrated in Fig. 2.

## Results

### Molecular results

**Figure 3.**
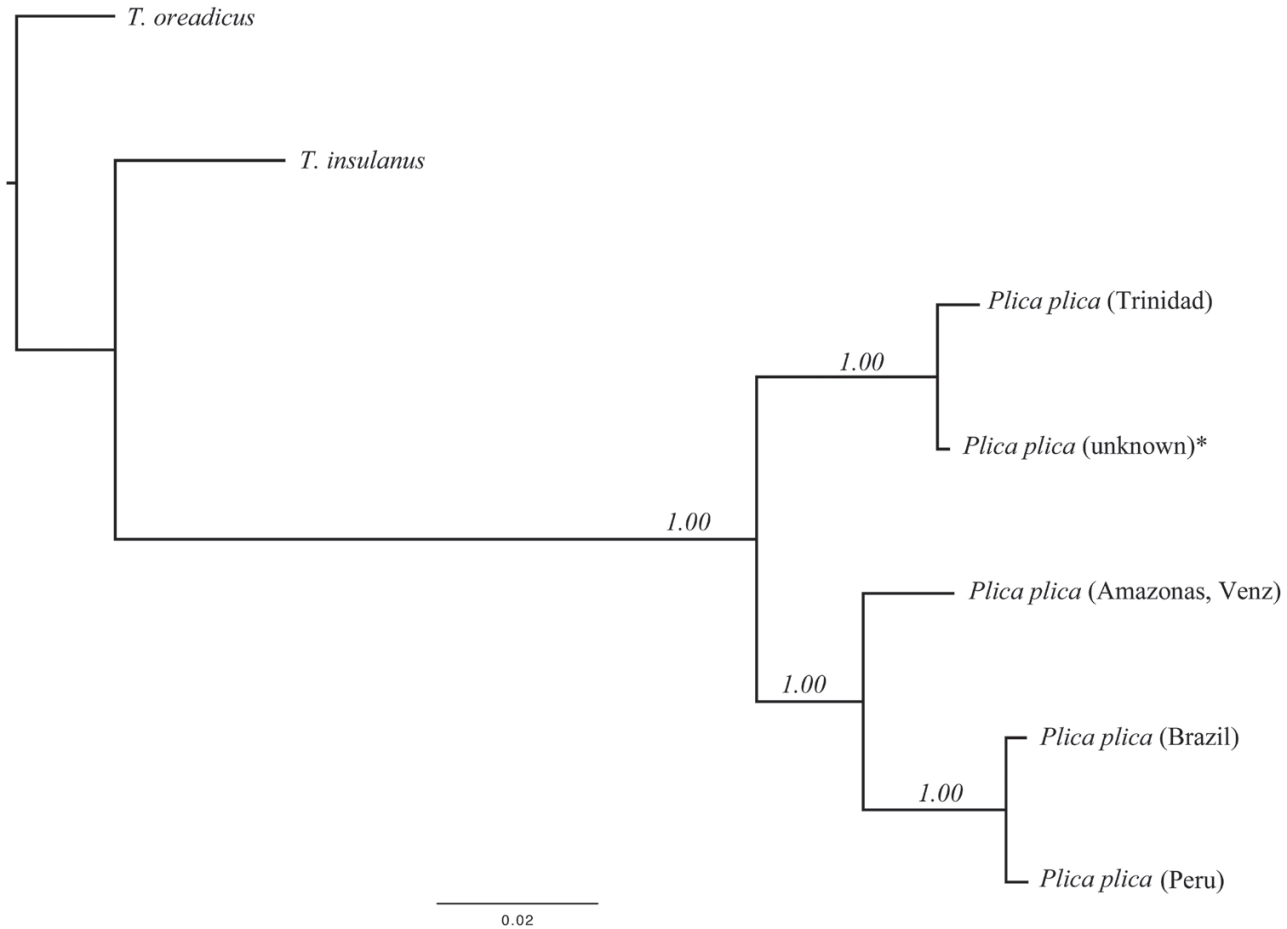
Bayesian Inference phylogram of all *Plica plica* species studied (12S and 16S rDNA partial genes, 715 bp). Values by nodes are the posterior probabilities recovered from the Bayesian Inference analysis. * *Plica plica* (unknown locality) was obtained from the pet trade.

The best-fitting model for the BI tree was the TIM2+I (**−** lnL=**−** 1567.2450, BIC= 3259.3633). *Plica plica* from Trinidad and one individual from the pet trade (unknown locality, probably collected on Trinidad or in adjacent Venezuela) showed little genetic variation (three substitutions). The Bayesian Inference consensus phylogram recovered three well-resolved monophyletic clades (BPP: 1) where *Plica plica* from Trinidad is monophyletic with the pet trade *Plica plica* and sister clade to all other *Plica plica* available from Genbank (Fig. 3). Genetic divergence estimates for the 12S and 16S rDNA partial gene sequences combined showed contrasting levels of differentiation between localities (Trinidad vs *Plica plica* from the pet trade: 0.04% and over 4% between Trinidad and all other *Plica plica* mainland localities) (Table 1).

### Morphological results

**Figure 4.**
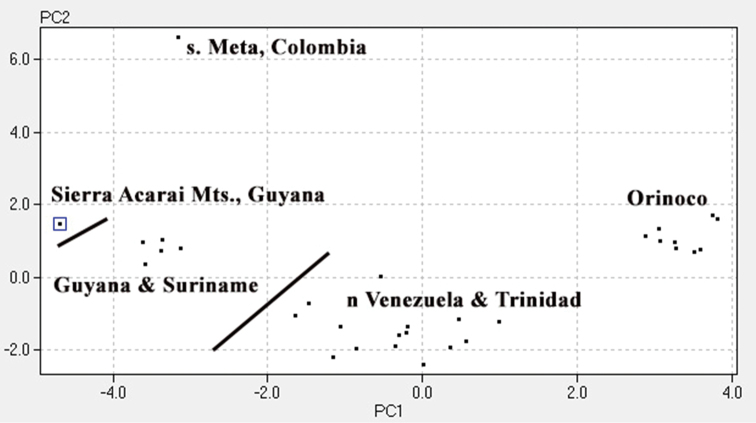
A PCA using 31 specimens labeled *Plica plica*. Scales around mid-body (28.20%), lamellae under fourth toe (13.74%), lamellae under fourth finger (11.09%), number of suboculars (7.14%), number of supraorbital plates (5.87%) accounted for 66.04% of the variation. Other traits used in the PCA included: dorsolateral fold, ventrolateral fold, scales between canthals, scales between nasals, loreals at canthal, scales between nasal and rostral, number of canthals, loreals keeled, upper labials, longest upper labial, lower labials, postmentals, ventral-post auditory tufts of spines, total tufts of spines, length of head, dorsal scale size, dorsal scale keels, dorsal pattern, and antegular fold.

For the morphological analysis we organized specimens geographically and compared them for 25 traits using a PCA (Fig. 4). The results support five morphologically distinct OTUs: one from the Caribbean Coastal Range and Trinidad; two from the Guyana Shield; one from southern Meta, Colombia; and one from the middle Orinoco drainage of Venezuela and Colombia. Table 2 compares the four species of *Plica* previously described to the four new species described here.

**Table 2.** A comparison of morphological traits for the species of *Plica*. Asterisk indicates that mainland Venezuela members of this species have 8–10 circumorbital scales, while Trinidad specimens have 10–12 circumorbital scales. Data for *Plica lumaria* from Donnelly and Myers (1991); for *Plica pansticta* from Myers and Donnelly (2001), for *Plica umbra umbra* and *Plica umbra ochrocollaris* from Avilla-Pires (1995). The ~ symbol denotes numbers taken from drawings. Abbreviations STN (spiny tufts on nape), SAAM (Position of spines on anterior margin of auditory meatus), SAB (scales around mid-body); SRN (scales between rostral and nasal). Numbers in parentheses after range indicate most frequent number.

Distribution	Caribbean Coastal Range	Acarai Mts, Guyana	Meta, Colombia	Suriname, Guyana, Venezuela	Middle Orinoco	Guaiquinima Tepui, s. Venezuela	Tepuis of northwest Venezuela	Widespread<br/> Amazonia	Bolivia
name	*Plica caribeana*	*Plica kathleenae*	*Plica medemi*	*Plica plica*	*Plica rayi*	*Plica lumaria*	*Plica pansticta*	*Plica umbra*	*Plica ochrocollaris*
n=	27	1	1	11	12	Literature	Literature	Literature	Literature
STN	2	2	2	2	2	2	2	0	0
Antegular fold	Complete	Interrupted	Complete	Interrupted	Interrupted	complete	Complete	Folds absent	Folds absent
Mite pockets	4	Absent	4	2	4	4	4	Absent	Absent
SAAM	Center	Ventral edge	Entire margin	Center	Ventral edge	Entire margin	Ventral half	None	None
SAB	92–125<br/> x=114.72<br/> SD=8.72	158	145	126–140<br/> x=133.75<br/> SD=4.57	181–202<br/> x=189.75<br/> SD=8.61	141–156<br/> x=146.2	143–164<br/> x=159	47–69<br/> x=60.5	42–56<br/> x=49.9
SRN	1–2	3	1/2	2–3	1	1	1–2	0–1	0–1
Lamellae 4^th^ finger	23–29<br/> x=26.2<br/> SD=1.89	29	32	25–28<br/> x=26.5<br/> SD=0.71	27–35<br/> x=29.1<br/> SD=2.39	20–24	23–30<br/> x=26.1	17–25	17–25
Lamellae 4^th^ toe	28–35<br/> x=31.26<br/> SD=1.92	35	41	31–36<br/> x=35.0<br/> SD=1.41	36–44<br/> x=39.40<br/> SD=2.88	27–33	31–39<br/> x=34.8	24–33	26–33
Scales on snout	Imbricate,<br/> keeled	Imbricate,<br/> pyramidal	Juxtaposed,<br/> conical, smooth	Imbricate,<br/> few keeled	Juxtaposed,<br/> flat, smooth	Imbricate, smooth	Imbricate,<br/> smooth	Variable	Variable
Intercanthals	6–8	8	8	7–9	7	~5	~7	~8	~5
Suboculars	5–6	6	6–7	5–7 (7)	4–5	1–5	7	1–9	~3
Circumorbitals	8–12*	12/12	11/9	10–13	7–10 (8)	~12	~11	7–11	~7
Internasals	5–6	8	5	7–8	5–6	~6	~6	7–11	7–11
Dorsal pattern	Green with black brown transverse markings	Uniform gray brown	Green, bold dark spots	Green with black brown markings	Dark mottling, white spots	Black w/yellow spots	Green gray with black blotches	Green dorsum	Green dorsum

### Available names

*Iguana chalcidica* Laurenti (1768: 48) has the type locality of “Gallaecia” (= northwest Iberian Peninsula) and the specimen was based upon an illustration in Seba (1734, 2: 65.5). Given that the type locality is in error, and the detailed scale arrangements and counts needed to identify the species are unavailable, this name is *nomen dubia*.

*Lophyrus panthera* Spix (1825: 10) was described from “*sylvis ad pagum Ecga*.” The holotype was in the Zoologische Sammlung des Bayerischen Staates, München, and destroyed during World War II. A fact recently reconfirmed by Franzen and Glaw (2007). Etheridge (1970) considered this speciesbased upon a brightly colored juvenile of *Plica plica* because ofthe description and the colored figure in Spix (1825: 11, pl 13, fig. 1). The type localityof *Lophyrus panthera* “Ecga,” is currently known as Tefé, Brazil (~03°21'S, 64°42'W). A specimen from Tefé was not located during this investigation. However we did examine specimens from locations within 500 km of Tefé, both localities are close to the Amazon River. One species has 95 scale rows at mid-body and 41 lamellae on the fourth toe; the other has 141 scales at mid body and 29 lamellae on the fourth toe. Therefore the *Plica plica* group in the Amazon basin is likely diverse and we take no further action on this name. That said, *Plica panthera* (Spix) is undoubtedly a valid name for a species that awaits rediscovery.

*Hypsibatus punctatus* Duméril & Bibron (1837, 4: 258) is based upon MNHN 2387 and it shares a similar number of scale rows around mid-body, subocular plates, and lamellae on fingers and toes with *Plica plica*. While the literature suggests *Hypsibatus punctatus* lacks a type locality (Etheridge 1970; Peters and Donoso-Barros 1970), Duméril and Bibron(1837) wrote that they thought the specimen originated from the same country as the species in the previous account (*Plica umbra* from Guyana or Suriname). Thus it is likely MNHN 2387originated from within the distribution of *Plica plica* as described here.

### The identity of *Plica plica* (Linnaeus,1758)

**Figure 5.**
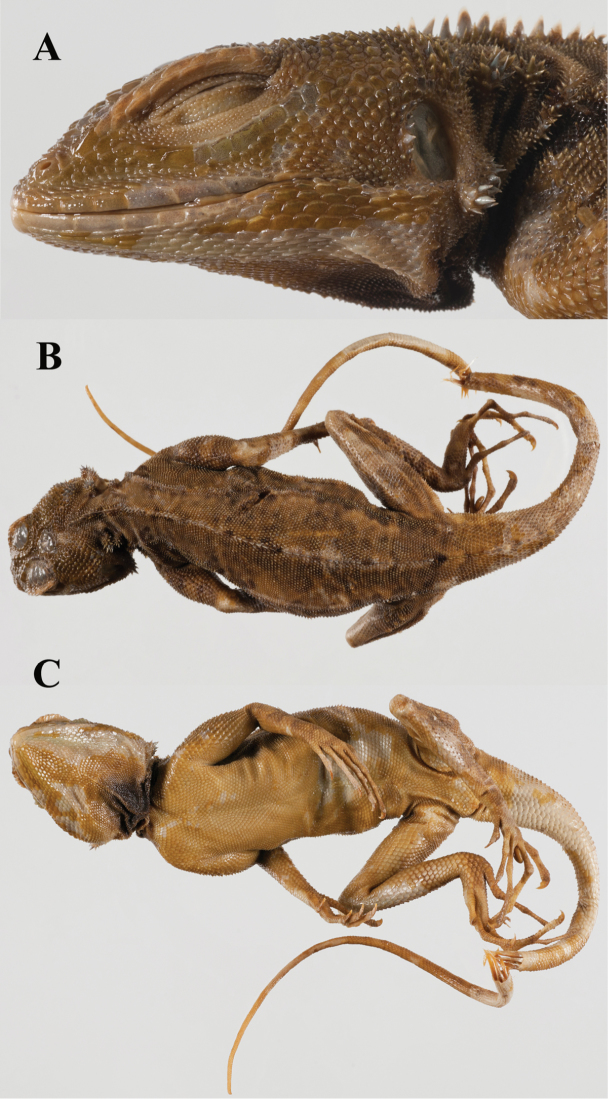
The holotype of *Lacerta plica* Linnaeus, 1758, NRM 112. Note the tuft of spines on the anterior edge of the auditory meatus (**A**); the indistinct spots on the dorsum (**B**) and the double dewlap fold (**C**). Photo credit: Sven Kullander.

Our comparison of Guiana Shield *Plica* and the lectotype (Fig. 5) to those from across the range are in close agreement with the description reported for Suriname specimens with 5–8 labials, usually six; mental in contact with 3–5 scales on posterior edge; dorsal crest well developed in males, slightly less so in females; ventrals 61–65; fourth finger with 24–28 lamellae; fourth toe with 31–36 lamellae.

**Figure 6.**
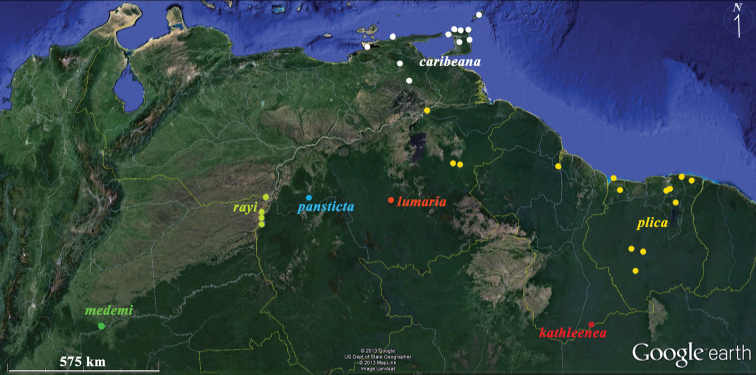
Distributions of *Plica plica* Group species discussed in this paper. Yellow – *Plica plica*; white *Plica caribeana* (because of scale not all Trinidad localities were plotted); red – *Plica kathleenae*; green – *Plica medemi*; light blue – *Plica rayi*; and the type localities of *Plica pansticta* and *Plica lumaria* are show in dark blue and orange respectively. A Google Earth map.

*Plica plica* occurs on the Guiana Shield in Suriname, Guyana, and Bolivar, Venezuela. It is also likely present in French Guyana. Three specimens (BMNH 67.5.4.1–2) from the island of Grenada were reported by Boulenger (1885), Barbour (1914) considered the locality data in error and suggested the specimens were from “New Grenada” (=Colombia). However, these specimens agree well with *Plica plica*. The adult male has 134 scale rows at mid-body; 28 lamellae on the fourth finger, 36 on the fourth toe; 4/5 upper labials; 7/6 suboculars; 13/13 semicircle scales. Thus, the specimens may represent a population now extirpated from the island, or an extant population that remains undiscovered (Henderson and Murphy 2012). The Bolivar, Venezuela specimens (KU167502–03) examined have a lower number of circumorbital scales (9–10) than specimens to the east which have 11–13 circumorbital scales. The locations from which *Plica plica* were examined as well as literature reports that reliably represent this species (Heyer and Barrio-Amorós 2009, Lasso 2009, Barrio-Amorós et al. 2011, Barrio-Amorós and Fuentes 2012) and are included in Fig. 6. For colors in life see Fig. 1c, d.

#### Plica caribeana sp. n.

http://zoobank.org/F06E2E69-0BD2-474F-827A-8D6F1FA1430B

http://species-id.net/wiki/Plica_caribeana

**Figure 7.**
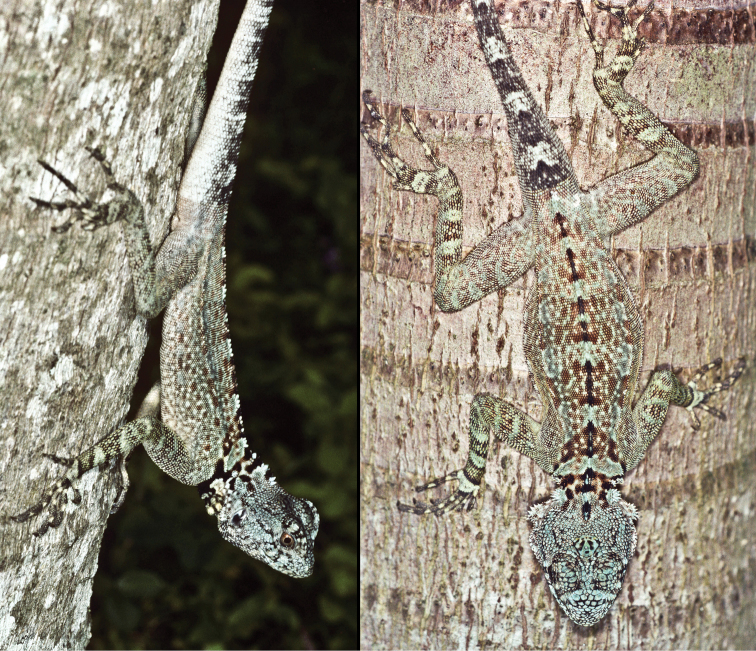
*Plica caribeana*, from the Arima Valley of Trinidad.

**Figure 8.**
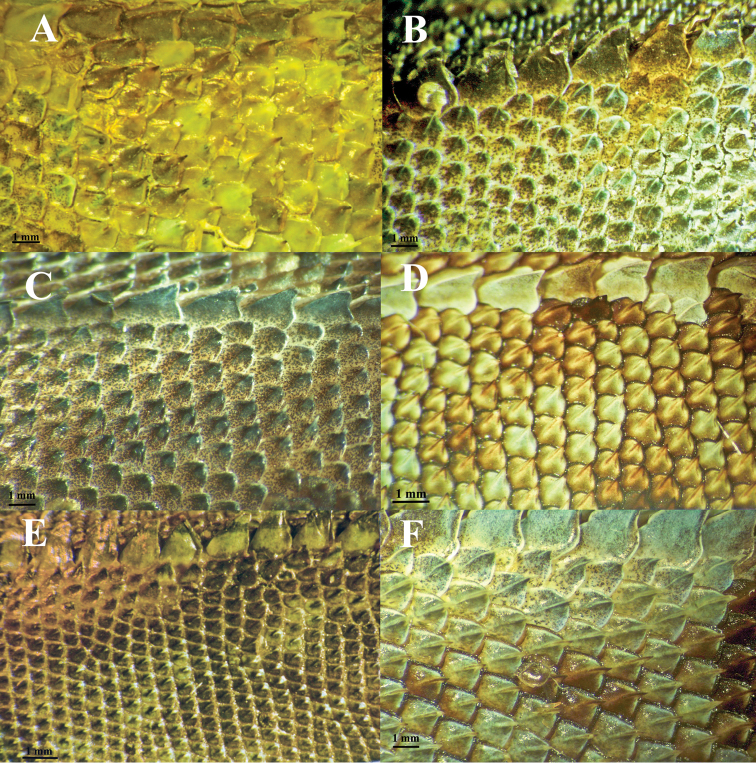
A comparison of dorsal scale morphology at mid- body near the dorsal crest. Anterior is to the left in all images. The scale bar shown in each image is one millimeter. Only large adult specimens were used for scale comparisons. **A** *Plica plica*, FMNH 128950**B** *Plica caribeana*, FMNH 49838 **C** *Plica kathleenae*, FMNH 30931 **D** *Plica medemi* FMNH 165207 **E** *Plica rayi*, FMNH 177925, and **F** is a *Plica* cf. *plica* from Amazonas, Venezuela, MCZ 101841.

**Figure 9.**
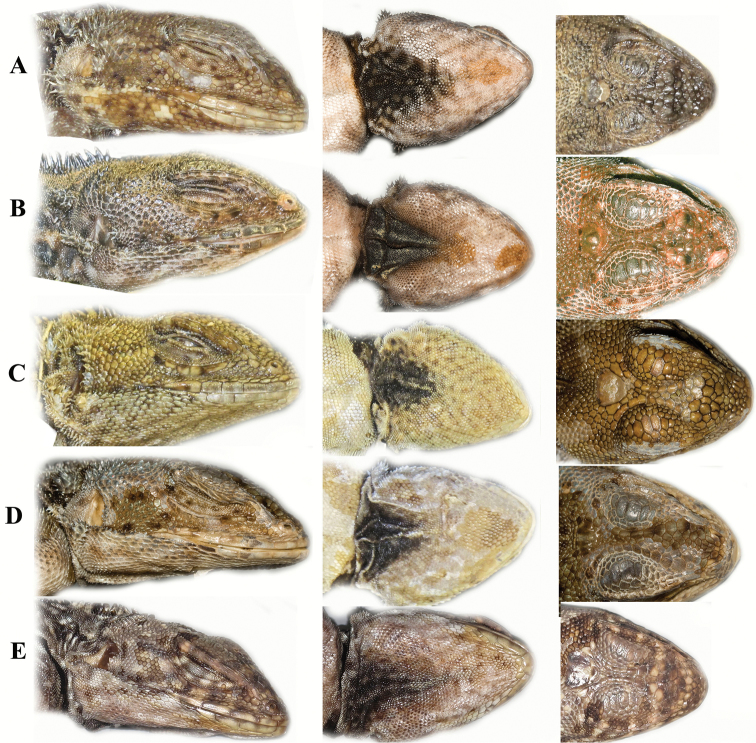
A comparison of head scalation for five species in the *Plica plica* Group found in northern South America. **A** *Plica caribeana* FMNH 49838 **B** *Plica kathleenae* FMNH 30931 **C** *Plica medemi* FMNH 165207 **D** *Plica plica* FMNH 128950 **E** *Plica rayi* FMNH 177926.

Caribbean treerunner Figs 7 , 8a , 9a

Hypsibatus agamoides – Court 1858: 440.
Uraniscodon plica – Boulenger 1885, 2: 180 [in part].
Plica plica – Burt and Burt 1931: 282 [in part].
Tropidurus plica – Frost 1992: 1 [in part].

##### Holotype.

FMNH 49838, an adult female, 110 mm SVL, 172 mm tail. Collected in 1947 by Frank Wonder, the Republic ofTrinidad & Tobago, Trinidad: San Rafael (~10°34'N, 61°16'W).

##### Other material.

Trinidad: Brickfield (~10°20'N, 61°16'W) 49834–35; Maracas (~10°45'N, 61°25'W) MCZ 60826; Mt. Harris (~10°30'N, 61°06'W) FMNH 49836; Nariva MCZ 60825; St. Annes MCZ 9002; San Rafael (~10°34'N, 61°16'W) FMNH 49837; Tucker Valley (~10°42'N, 61°36'W) FMNH 40448; no specific locality FMNH 25014; Cuesa River, Chagaramas (~10°43.319N, 61°38.6'W) UWIZM 2012.27.56. Venezuela: Sucre, Guaranunos (~10°32'N, 63°06'W) KU167497–98, 167500–3; Cumana, Muchorapo (~10°27'N, 64°10'W), KU117088. Sucre, Chacaracual (~9°51'N, 63°42'W) MCZ 43861–65, 81186–89; Monagas: MCZ 88185.

##### Diagnosis.

A *Plica* with dorsal scales in 92–125 (usually 110–125) rows at mid-body; scales on snout imbricate and keeled; dorsolateral and ventrolateral folds well developed; head length 21–22% of the SVL; one longitudinal dewlap fold; and a dorsal pattern of black and green transverse bands. *Plica plica* has 126–140 dorsal scale rows at mid body and two longitudinal dewlap folds. The middle Orinoco species, *Plica rayi* sp. n. has 180–202 scale rows at mid-body; flat, juxtaposed scales on the snout; and the ventrolateral fold is weakly spinose. The Sierra Acarai Mountain's (Guyana) *Plica kathleenae* sp. n. has 158 scale rows around mid-body; head 29% of SVL; and lacks gular mite pockets (all other species have them). The species from southern Meta, Colombia *Plica medemi* sp. n., has 145 scale rows at mid-body, and a dorsal pattern of six rows of small, bold, dark, irregular spots. The two species, associated with the Venezuelan tepuis (*Plica lumaria* and *Plica pansticta*) have a higher number of scale rows at mid-body (141–164) and smooth scales on the snout.

##### Description of holotype.

Rostral band-like (much wider than tall), contacts five post-rostral scales; nasals positioned over first labial, separated from upper labials by 1–2 scales, separated from each other by six scales; scales on snout imbricate, keeled, in regular rows, with asperities; loreals in seven rows (some keeled); canthals single, contacts three loreals, separated by eight inter-canthals scales; circumorbital scales 11/12, keeled, smaller posteriorly; separated from occipitoparietal by small scales; occipitoparietal broader than long; subocular plates 5/5, with serrated keel; upper labials 5/5, fifth is longest; supraciliaries in three layers, eight scales per layer; auditory meatus has a cluster of spiny scales on anterior margin, none on posterior margin, ventral fold has a cluster of spiny scales; two tufts of spines on neck, anterior tuft largest; antegular and gular folds complete, dewlap fold originates from the antegular fold; four mite pockets present, two under each fold; mental rounded, contacts four postmentals; lower labials 6/6; scales on throat small, quadrangular and smooth; dorsal crest well developed onto tail; dorsolateral fold well developed to thigh; ventrolateral fold present along length of body; scales around mid-body 125; ventrals 53; dorsal surfaces of limbs covered with mucronate scales, ventral surfaces covered with larger, smooth scales; fourth finger with 29 lamellae; fourth toe with 32 lamellae; feet 37% of SVL; tail depressed at base, cylindrical distally.

In alcohol, a uniform brown gray dorsum; tail with alternating, indistinct dark and pale bands; forearms and lower legs also with indistinct bands; chin spotted, black gular center; venter of body and tail cream.

##### Variation.

Rostral makes contact with five or six post-rostral scales; nasals separated from each other by 5–7 scales; canthal single or double, in contact with 2–4 loreals, separated by 6–8 inter-canthal scales; circumorbital scales 11 or 12 in Trinidad and northern Venezuelan populations, 8 to 10 in Bolivar, Venezuela populations; upper labials usually four, rarely five; loreal region has 6 –9 rows of weakly keeled loreals between canthal and upper labials; suboculars usually five, rarely six; lower labials 5–6, bordered by 3–5 rows of slightly enlarged scales; dorsal scales in 92–125 rows around mid-body (usually 110–125); dorsal crest well developed in males, less so in females.

Color in life (Fig. 7), head mostly green with black markings on supraciliaries, a black postorbital stripe and indistinct black bars on labials; dorsum mostly green with 4–6 transverse brown bands; vertebral crest mostly black but interrupted with green between the brown bands; limbs and feet with alternating green and brown bands; tail with alternating black and green bands; ventral surface mostly uniform white with brown mottling.

Body and tail sizes. Female’s SVL 105–121 mm (n=6, x=98.8. SD=18.22); undamaged tails 132–205 mm (n=5, x=170.6, SD=33.83). Male’s SVL 81–121(n=12, x=104.17, SD=12.04); undamaged tails (n=7) 132–205 mm (x-=170.6, SD= 32.63). Hind feet average about 39% of the SVL.

##### Etymology.

Named for its Caribbean Coastal Range distribution.

##### Distribution.

Eastern Coastal Range (Cordillera de la Costa Oriental) of Venezuela south into Bolivar; Trinidad, the Bocas Islands (Huevos, Monos, and Gaspar Grande); Tobago (Murphy and Downie 2012).

##### Ecology.

*Plica caribeana* is a forest and forest-edge species most frequently observed on tree-trunks, rock walls, walls of caves, and buildings. They often occur in small colonies of 6–15 individuals, usually positioned with head downward, but slightly raised off the substrate. They are ambush insectivores feeding on ant columns, beetles, cicadas, spiders, and other arthropods. Females are reported to lay clutches of two eggs; the smallest juvenile measured for this study was 47 mm SVL with a damaged tail; this species is known to be preyed upon by the snake *Siphlophis compressus* (Boos 1977; Murphy 1997). Note that Beebe’s (1944) comments on *Plica plica* may be partially applicable to this species.

#### Plica kathleenae sp. n.

http://zoobank.org/D1F2C377-1FEB-4F02-A017-0C5A4D4AFE0C

http://species-id.net/wiki/Plica_kathleenae

Kathleen’s Treerunner Figs 8b , 9b

##### Holotype.

FMNH 30931. An adult male, 124 mm SVL, tail damaged. Collected by Emmet Reid Blake on the Sewell Avery British Guiana Expedition, in September-October, 1938; Guyana, Boundary Camp, Itabu Creek headwaters (~1°42'N, 57°55'W) in the Sierra Acarai Mountains near the Brazilian border, at an elevation of about 549 m).

##### Diagnosis.

A *Plica* with dorsal scales in 158 rows at mid-body, 6 suboculars; scales on snout mostly imbricate and slightly pyramidal with asperities; head length 29% of the SVL (other species have heads 17–23% of the SVL); gular fold complete, antegular fold incomplete; dewlap originates in the space between the two folds; throat folds in this species are relatively shallow and do not form the mite pockets seen in other species. *Plica plica* has fewer scale rows at mid-body (126–140) as well as complete antegular folds. *Plica caribeana* sp. n. has 92–125 scale rows at mid-body, and scales on snout are keeled and imbricate. *Plica medemi* sp. n. has 145 scale rows at mid-body; seven suboculars; head length of 23% of the SVL. *Plica rayi* sp. n. has 181–202 scale rows at mid-body; and flat, juxtaposed scales on the snout. *Plica lumaria* and *Plica pansticta* have smooth imbricate scales on snout and one scale between nasal and rostral.

##### Description of holotype.

Rostral in contact with six post-rostrals; nasals separated from rostral by three scales, internasals eight; scales on snout imbricate, slightly pyramidal, with one or two asperities; canthals single; separated by eight inter-canthal scales; circumorbital scales 12, separated from the occipitoparietal by a row of small scales; occipitoparietal slightly broader than long; supraorbital plates 5/6, separated from the superciliaries by two rows of small scales; superciliaries three layers with about eight scales in each layer; suboculars 7/6, first is largest (fourth almost as large), with serrated keels; upper labials five, fifth is longest; six lower labials; loreal scales slightly keeled, in 8–10 rows between canthal and upper labials; auditory meatus with a spiny tuft on anterior margin, rest of margin with smaller mucronate scales; ventral flap with large tuft of spines; two clusters of spiny tufts on nape; mental small, in contact with four tiny post mental scales; gular fold complete; antegular fold incomplete; dewlap originates anterior to gular fold, transects the antegular fold, mite pockets absent; dorsal crest well developed on anterior body; scales around mid-body 158; ventrals 63; limbs well developed and covered with keeled mucronate scales above, and smooth scales below; lamella under fourth finger 29; lamellae under fourth toe 35. Feet 33% of the SVL.

In alcohol, dark brown dorsum with an irregular row of dark spots with light centers on each side of the mid-line, some of the dark spots are surrounded by smaller white spots; small white spots are also on the front limbs. There is a narrow dark collar that extends laterally. Forelimbs and hind limbs have transverse bands.

##### Etymology.

Named in honor of Kathleen Kelly, Division of Amphibians and Reptiles, Field Museum of Natural History, for her interest and effort on behalf of herpetology.

##### Distribution.

Known only from the type locality in the Acarai Mountains of Guyana.

##### Ecology.

Nothing is known about the ecology of this species, but see the discussion.

#### Plica medemi sp. n.

http://zoobank.org/C2FCD890-B516-44F5-B728-46AB3F4CEA16

http://species-id.net/wiki/Plica_medemi

Medem’s Treerunner Figs 8c , 9c

##### Holotype.

FMNH 165207 an adult male, 112 mm SVL, with a damaged tail. Collected by Fredrico Medem in 1957. Colombia, Meta, Lower Guayabero, Angostura No. 2, Cerro de las Pinturas (~2°34'N, 72°51'W).

##### Diagnosis.

A *Plica* with dorsal scales in 145 rows at mid-body; scales on snout juxtaposed, smooth and slightly domed; dorsolateral and ventrolateral folds well developed and extend to groin; entire anterior margin of auditory meatus is lined with spiny scales (no distinct cluster of spines); 41 lamellae on fourth toe. It has a green dorsum with black spots, and an orange head and lacks the transverse bands present in other members of the genus. *Plica plica* has 126–140 dorsal scale rows at mid body, imbricate, keeled scales on the snout; and 31–36 lamellae on the fourth toe. *Plica rayi* sp. n. has 180–202 scale rows at mid-body; juxtaposed and flat scales on the snout; and the ventrolateral fold is barely visible. *Plica kathleenae* sp. n. has 158 scale rows around mid-body, and a cluster of spiny scales on the anterior margin of the auditory meatus. *Plica medemi* sp. n. differs from *Plica lumaria* and *Plica pansticta* in having more lamellae on the fourth finger and fourth toe (32 and 41 respectively), supracilliaries directed dorsally as opposed to laterally; an exceptional spiny texture to its scales, and a distinctly different pattern.

##### Description of holotype.

Rostral band-like, in contact with five post-rostrals; scales on snout juxtaposed, smooth, but bulge to form slight domes, asperities few in number; nasals separated from rostral and upper labials by 1/2 scales; five internasals; nine inter-canthals; circumorbitals 11/9, moderately distinct posteriorly, rounded, smooth, medially; six supraocular plates separated from circumorbitals and superciliaries by small scales; occipitoparietal subtriangular, about as wide as broad; canthal short, sharply keeled, contacts two loreals; superciliaries in three layers of 7–9 upturned, keeled scales; loreal region has 7/8 rows of scales between canthal and upper labials (counted diagonally); subocular plates 7/6 each sharply keeled, the first is the longest; five elongated upper labials, the last is the longest; five lower labials similar to upper labials, bordered below by two or three rows of larger scales; mental sub-triangular contacts three small post-mentals; antegular fold complete; gular fold incomplete; the dewlap originates from the antegular fold; the folds form four, lateral mite pockets (two under each fold); gulars small, imbricate, smooth anteriorly, decreasing in size posteriorly, keeled near the transverse gular fold; mid-dorsal crest composed of a row of enlarged spiny scales extending from occiput to proximal half of tail; two tufts of enlarged, spinose scales on side of neck; spiny scales line the entire anterior edge of auditory meatus, a cluster of spinose scales absent; one tuft of spiny scales on auricular flap that covers most of its surface; dorsal scales around mid-body 145; ventrals 44; dorsolateral fold well developed from shoulder to thigh; ventrolateral fold present; caudal scales similar to body scales above and below; scales on upper limbs keeled, very spinose, and imbricate; ventral limb scales mostly smooth, imbricate; scales on dorsal surface of digits keeled ending in a stout spine. Lamellae on fourth finger 32, on the fourth toe 41, each has a median keel ending in a stout spine; hind feet about 34% of SVL.

In alcohol, the head is orange the dorsum of the body is green with dark, bold spots, no transverse blotches on the dorsum. The collar is a network of dark pigment with light colored area surrounded by darker pigment. Upper surfaces of the limbs are similarly patterned with bold spots, but transverse bands are present distally. The venter of the chin is spotted with a black area of pigment around the dewlap; the venter of the body is a uniform cream.

##### Etymology.

The lizard is named in honor of Colombian herpetologist Fredrico Medem.

##### Distribution.

Known only from the type locality at Angostura No. 2, Cerro de las Pinturas, Lower Guayabero, and Meta, Colombia.

##### Ecology.

Nothing is known about the ecology of this species, but see the discussion.

#### Plica rayi sp. n.

http://zoobank.org/6DE2FD0E-8AA0-4563-8858-B11CFF86277D

http://species-id.net/wiki/Plica_rayi

**Figure 10.**
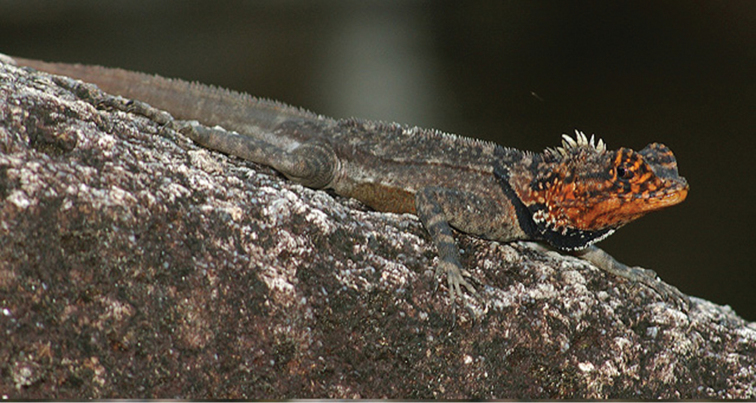
*Plica rayi*. A male in breeding coloration. Photographed at Tobogan de la Selva, Puerto Ayacucho. Photo credit Zelimir Cernelic.

Ray’s Treerunner Figs 8e , 9e , 10

Plica plica – Etheridge 1970: 242 (in part).

##### Holotype.

FMNH 177926 a male. Collected by Gary Myers in 1962. Venezuela, Amazonas, Puerto Ayacucho (~05°39'N, 67°38'W), on the Orinoco River.

##### Other material.

Colombia– Vichada, Puerto Carreno, (~6°08'N, 67°26'W) MCZ 150179–81; Venezuela – Puerto Ayacucho FMNH 177924–34, MCZ58335–36, 160222–24.

##### Diagnosis.

A *Plica* with greatly reduce mucronate scales, the two clusters on the neck reduced to small knobs; in overall appearance this lizard has a smooth external texture; 182–202 scales around mid-body (more than any other *Plica* species); seven subocular plates; scales on snout juxtaposed and flat; nasal scales separated from rostral by a single scale; spiny scales around auditory meatus greatly reduced or completely absent; lamella under fourth toe 36–45 (more than any other *Plica* species). Males have a well-developed dorsal crest that extends onto the tail; females have this crest greatly reduced. *Plica caribeana* sp. n. has 125 or fewer scales around mid-body and imbricate, keeled scales on the snout. *Plica kathleenae* sp. n. has 158 scales at mid-body and imbricate scales on the snout. *Plica medemi* sp. n. has 145 scale rows at mid-body and well developed spines on the anterior margin of the auditory meatus. *Plica plica* has 140 or fewer scales around mid-body; keeled, imbricated scales on the snout, and has well developed spiny scales. The two tepui associated species (*Plica lumaria* and *Plica pansticta*) have fewer scale rows around the body (141–164), imbricate scales on the snout and fewer lamellae on the toes.

##### Description of holotype.

FMNH 177926, male, 103 mm SVL, 175 mm tail. Rostral broader than tall, with small asperities, makes contact with five post-rostral scales; nasals small, separated from upper labials by one scale, separated from each other by five scales; scales on snout, juxtaposed, flat with round asperities; canthal single, makes contact with three loreals, separated by seven inter-canthal scales; circumorbital scales weakly keeled, well developed, nine on each side, and maintain their size posteriorly; occipitoparietal broader than long, in direct contact with circumorbital scales; upper labials 4/4, fourth is the longest; lower labials five, bordered by four rows of slightly enlarged scales below; loreals in 8/9 rows between canthal and upper labials, scales weakly keeled; dorsal crest on nape and extends to about mid-tail, scales relatively small, largest spines close to occipital region; dorsolateral fold well developed on anterior body extends passed thigh, ventrolateral fold barely discernible in preserved specimens; dorsal scales exceptionally small; scales around mid-body 190; ventrals 80; auditory meatus with small cluster of reduced spines on anterior margin, none on posterior margin; ventral flap has few spines on margin; two small tufts of spines on neck, reduced to almost smooth knobs, anterior tuft larger than posterior; gular fold complete; antegular fold incomplete; dewlap originates on the gular fold; mental rounded, in contact with four scales on posterior edge; scales on throat small, smooth, subtriangular; ventrals larger than dorsals, imbricate, smooth; fourth finger with 27 lamellae; fourth toe with 37 lamellae; feet about 35% of SVL; tail laterally compressed at base and along most of its length.

In alcohol, dark transverse bands on the snout and back of head, as well as a well-formed shoulder stripe that extends onto neck and throat. Dorsum brown gray with light spots; tail with alternating, indistinct dark and pale bands; forearms and lower legs also with indistinct bands; chin spotted, black gular center; venter of body and tail cream.

##### Variation.

Rostral may or may not have asperities; nasals separated from upper labials by one or two scales, separated from each other by 5–6 scales; circumorbital scales usually 8–9 (rarely 7 or 10), keeled, and may become slightly smaller posterior; loreal region has 7–9 rows between canthal and upper labials, scales weakly keeled; suboculars 4–5, usually four; mental rounded, in contact with 3–4 scales on posterior edge; lower labials 4–6, usually five; scales around mid-body 181–202; ventrals 80–93; fourth finger with 27–35 lamellae; fourth toe with 36–45 lamellae. Preserved specimens show poorly defined dorsal crests, males have greatly enlarged spines in the occipital region, these are barely discernible in females; dorsolateral and ventrolateral folds not obvious in preserved specimens; in life these folds are quite distinct based on Fig. 10.

Body and tail. Female’s SVL 66–106 (n=8, x=79.13, SD=17.21); undamaged tails 134–175 (n=4, x=148.50, SD=15.79). Male’s SVL 85–105 (n=3, x=96.00, SD=8.92), undamaged tails (n=1) 143 mm. Feet 35–39% of SVL; tail depressed at base, becomes more cylindrical distally. Individuals measured for this study ranged from 55–106 mm SVL, and had unbroken tails that were up to 2.1 times the SVL.

In alcohol, a uniform brown gray dorsum; dorsum has some traces of transverse bands, but white spots numerous on some individuals; tail with alternating, indistinct dark and pale bands; forearms and lower legs also with indistinct bands; chin spotted, black gular center; venter of body and tail cream.

In life breeding males have a red-orange face and throat with about five irregular black brown markings extending from the supraorbital crest to the upper labials and some extend onto the throat; crown and face otherwise brown. A black nape blotch extends from the dorsal crest onto the throat where it widens and contains some white pigment; the body is brown-black with indistinct white and black markings; limbs with indistinct bands. Females are brown black with the head slightly darker in color than the body, and a black gular blotch at the anterior edge of the dorsolateral fold; upper forelegs with light colored mottling, lower forelegs with indistinct bands.

##### Etymology.

This lizard is namedin honor of Ray Pawley, former Curator of Reptiles at Brookfield Zoo, for his lifelong interests and work on amphibians and reptiles. Suggested common name: Ray’s Treerunner.

##### Distribution.

Known from two localities along the Orinoco River:Puerto Ayacucho, Amazonas, Venezuela and Puerto Carreno, Vichada, Colombia. The distance between these two locations is about 65 km. They have also been observed at Tobogan de la Selva (~5°23'13"N, 67°37'0"W) and Raudal de Danto at Autana (4°48'N, 67°29'W, 89 m asl).

##### Ecology.

*Plica rayi* is associated with granitic rainforests. It is very abundant in rocky areas; tobogans are granite slabs used as refugia by the lizards. In May, coinciding with the initiation of rains, males have a bright red-orange head coloration not observed in other months (July, September, or December). At night they sleep vertically with the head facing the sky (César Barrio-Amorós, personal communication).

## Discussion

Molecular and morphological data support the hypothesis that *Plica plica* is a complex of cryptic species that are concordant with features of South American geography. We identify *Plica plica* (Linnaeus, 1758), *Plica kathleenae* sp. n., and *Plica rayi* sp. n. as Guiana Shield endemics or near endemics. Hoogmoed (1979) considered 23 of the 65 (35%) Guyana lizards known at the time to be endemic and he viewed the Acarai Mountains as one of the most important refugia for the forest herpetofauna. More recently, Avila-Pires (2005) reported 118 lizards on the Guiana shield, 52 (44%) were listed as endemics. *Plica kathleenae*, *Plica plica* and *Plica rayi* can now be added to that list.

The four new species described here all appear to be associated with areas of endemism or specific features of the northern South American landscape.

*Plica caribeana* is associated with the Eastern Coastal Range bioregion of Rivas et al. (2012), as well as Trinidad and Tobago. Other lizards restricted to this region include: *Ameiva atrigularis*, *Anadia pariaensis*, *Euspondylus monsfumus*, *Gonatodes ceciliae*, *Gonatodes seigliei*, *Riama rhodogaster*, and *Riama shrevei*. Mitochondrial genetic divergence for gene fragments in Squamata (16S: 0.45% and 12S: 0.5%, per million years, Carranza et al. 2004; Poulakakis et al. 2005, respectively) would suggest a genetic divergence between the mainland *Plica plica* Group and *Plica caribeana* in the Late Miocene. This would have preceded the separation of Trinidad from the mainland (Audemard and Audemard 2002; Moretti et al. 2007; Persad 2009).

*Plica kathleenae* is known only fromthe type locality in the Acarai Mountains. The Itabu Creek, is a small tributary entering the New River from the south near its headwaters, and provides a route into the Acarai Mountains which separate the Amazon drainage from that of the Essequibo and Courantyne drainages (Blake 1941). The area was the subject of a Rapid Biological Assessment in 2006 and the team documented 34 species of reptiles, including “*Plica plica*.” The report suggests the area contains a rich herpetofauna and is considered to be a center of endemism (Señaris et al. 2008). The distribution and natural history of this lizard remains to be determined.

*Plica medemi* is known only from the type locality in La Macarena National Park at the base of the Colombian Andes, an area transitional between Guyana and Amazonia. Kattan et al. (2004) found the Macarena Range to have the most diverse fauna per unit of area in the Colombian Andes with much of the fauna associated with the Guiana Shield (based upon butterflies, frogs, birds, bats and rodents). It is unclear if this *Plica* is a Macarena endemic, or is more widespread.

*Plica rayi* is known from the western edge of the Guiana Shield along the middle Orinoco. The Orinoco-Negro white sand forest bird area is nearby and considered a center for endemic bird species. The forest is mostly undisturbed primary lowland humid forest up to about 500 m that follows rivers into the middle Orinoco watershed (BirdLife 2012). Groger and Huber (2007) describe the Puerto Ayacucho area as a true center of endemism for the flora due to an overlap in the northern and southern units of the inselberg vegetation. Additionally, Barrio-Amorós et al. (2010) described a new species of frog in the genus *Anomaloglossus* (Dendrobatidae) from this region and considers the Puerto Ayacucho biodiverse but poorly known.

There is at least one unresolved Venezuelan species of *Plica* from the Amazonas portion of Venezuela. It has a relatively low dorsal scale row count (113–114), two tufts of spines on the auricular fold, and four subocular plates. The survey of the specimens examined here suggests Peru has at least three distinct species, Ecuador has at least one other, and Brazil has several more, one of which will be revalidated as *Plica panthera* (Spix). Distinguishing the species present in the *Plica plica* Group will help clarify the evolution of the forested South America landscape and expand our knowledge of tropidurid biogeography.

## Supplementary Material

XML Treatment for Plica caribeana

XML Treatment for Plica kathleenae

XML Treatment for Plica medemi

XML Treatment for Plica rayi

## Appendix 1

Specimens examined but not assigned a name. Brazil: FMNH 83595, MCZ 92750. Colombia: MCZ150179−81. Ecuador: FMNH 42502−4, MCZ 37268. Peru: FMNH 45493−5, 56011-20 56104, 83305, 81451−2, 109821, 109826−27, 134427, 228259. Venezuela: MCZ 23166, 101841.

## Appendix 2

**Table:** Factor loadings of 25 meristic and morphometric traits. Eigenvalues and percentage of variance explained by the respective components are at the bottom of the table. Abbreviations: scales around mid-body (SAB); lamellae on 4th finger (LAMF); lamellae on 4th toe (LAMT); number of suborbital plates (SUBO); number of supraorbital plates (SPRO); dorsolateral fold (DF); ventrolateral fold (VF); intercanthals (IC); Internasals (IN), scales separating nasal from rostral (NR); loreals contacting canthal (LC); loreals keeled (LK); upper labials (UL); longest upper labial (LUL); lower labials (LL); postmentals (PM); spiny tuffs on ventral auditory fold (VTS); total tuffs of spines (TT); dorsal scale size (DSS); keels extend over scale apex (KOA); head length (HL); dorsal scales broad (DSB); antegular fold (AF);

	PC1	PC2	PC3
SAB	0.266	0.316	0.164
LAMF	0.224	0.336	0.094
LAMT	0.201	0.175	-0.008
SUBO	0.297	0.065	0.064
SUPRO	0.227	-0.126	-0.188
DF	0	0	0
VF	0.325	-0.196	-0.124
IC	-0.244	0.059	0.13
IN	-0.217	-0.064	0.256
NR	-0.223	0.208	0.353
CAN	0	0	0
LC	0.045	-0.313	0.328
LK	0	0	0
UL	-0.269	0.227	0.07733
LUL	-0.268	0.1943	0.059
LL	-0.197	0.123	0.045
PM	0.077	-0.072	0.102
VTS	-0.074	-0.025	0.106
TT	-0.074	0.319	-0.451
KOA	0.325	0.196	0.124
HL	-0.109	0.122	0.221
DSB	-0.074	0.319	-0.451
DSS	0	0	0
DP	0.326	-0.159	-0.001
AF	-0.04	0.392	0.294
Eigenvalues	7.52	3.44	2.72
Variance Explained	30.08	13.78	10.89
F (ANOVA)	45.64	5.22	5.33
Significance (ANOVA)	<0.01	<0.01	<0.01
DF	24	24	24
